# Analysis of RNA yield in extracellular vesicles isolated by membrane affinity column and differential ultracentrifugation

**DOI:** 10.1371/journal.pone.0238545

**Published:** 2020-11-06

**Authors:** Gilberto Gutiérrez García, Gabriela Galicia García, Jessica Zalapa Soto, Andrea Izquierdo Medina, Mariana Rotzinger-Rodríguez, Gustavo Antonio Casas Aguilar, Cynthia Paola López Pacheco, Álvaro Aguayo, Maria Montserrat Aguilar-Hernandez

**Affiliations:** 1 Instituto Nacional de Ciencias Médicas y Nutrición Salvador Zubiran, Mexico, Mexico; 2 FES Zaragoza, Universidad Nacional Autónoma de México, Mexico, Mexico; 3 Facultad de Medicina, PECEM, UNAM, Mexico, Mexico; 4 Facultad de Medicina, Universidad Anahuac, Mexico, Mexico; 5 Laboratorio Nacional de Citometria de Flujo, Investigaciones Biomédicas UNAM, Mexico, Mexico; 6 Catedra CONACYT para Jóvenes Investigadores; Harvard Medical School, UNITED STATES

## Abstract

Extracellular vesicles (EV) have attracted much attention as potential biomarkers due to their protein, RNA and other nucleic acid content. The most common method used for EV isolation is differential ultracentrifugation (DU), however given the DU technical difficulties, other more practical methods have surged, such as membrane-affinity column commercial kits. Here, we assessed one commercial kit in terms of EV recovery and EV-derived RNA yield and compared it with a DU protocol. Our data shows that the commercial kit preparation results in a lower count of EV-like structures and a reduced expression of EV markers when compared to DU samples. Thus, apparently suggesting that the commercial kit had a lower EV yield. However, these findings did not reflect on RNA yield, which was greater with the commercial kit, even after an enzymatic treatment with proteinase K and RNAse A. We conclude that the kit has a higher EV-derived RNA yield in comparison to our DU protocol, suggesting that it may be the method of choice for RNA sequencing purposes.

## Introduction

Extracellular vesicles (EV) is a generic term used to define particles delimited by a lipid bilayer that originate from the cell [1,2]. Classification of EV has been a source of debate, since many definitions have been proposed. One of the earliest and most used nomenclatures divided EV into two main subtypes: microvesicles and exosomes [3,4]. They were differentiated by size and mechanism of biogenesis. Exosome definition included an endosomal origin and a size of between 50–150 nm while particles formed by budding from the plasma membrane and with a broader size distribution (100–1000 nm) were classified as microvesicles [5,6]. MISEV 2018 newer consensus has proposed that EV definitions should be based more on the size and biophysical properties of EV, since it is difficult to determine the biological origin of vesicles [1].

EV have almost ubiquitous presence throughout distinct biofluids [7–9]. Therefore, they have been investigated as potential biomarkers [10–12]. EV have taken great relevance in the biomedical field due to their cargo in proteins [13], lipid [14] and nucleic acids [15,16] that resemble those of the cell of origin thus giving them a distinctive molecular signature. One of the most studied EV-associated biomarkers are short RNAs [17–19]. It has been shown that EV have a characteristic profile of small RNA lacking ribosomal RNA peaks [20].

EV show several characteristics that make them strong candidates for biomarker research. Firstly, they are present in biofluids that are easier and safer to obtain than tissue biopsies, particularly in sites of difficult access like the central nervous system. Moreover, they contain RNA and DNA that can aid in the diagnosis, disease monitoring and early detection or relapse of diseases [21,22]. Several studies have shown promising results regarding EV derived RNA as a potential biomarker, mainly in the cancer field [23,24]. However, before EV-derived RNA can be used in the clinical setting, there is still a need for additional studies regarding not only its biology and function but also the technical aspects of RNA isolation and characterization, especially since many different methods are available [25].

To further study EV-derived RNA as a biomarker, a series of recommendations have to be considered: the chosen isolation method has to result preferentially in a high yield of EV and EV-derived RNA to use in downstream experiments such as sequencing [1]; if the main interest is to work with RNA enclosed in vesicles, then a treatment with proteinase K and RNAse A before RNA extraction will degrade non-vesicle RNA [26–29]; the isolation method has to work for culture supernatant and plasma samples considering that they both have different biophysical properties and sometimes analysis of both fluids is necessary as they provide complementary information [30]; finally, it is desirable that the method co-isolates few lipoproteins, particularly high density lipoprotein (HDL), as this is a miRNA carrier that can contaminate the exosomal RNA repertoire [31,32].

The most widely used EV isolation method is Differential Ultracentrifugation (DU), a technique that separates particles based on their buoyant density [33]. This method is low cost and lacks additional chemicals, although it is time-consuming and carries complexity due to the many factors that can alter the EV yield [34]. Other less time-consuming methods have been developed, such as membrane-affinity column commercial kits, which could be more useful in the clinical setting as they are faster and easier to perform [35,36]. However, it still must be demonstrated that membrane-affinity column commercial kits obtain higher EV recovery and EV-derived RNA yield in comparison to traditional isolation methods.

There are only two studies that have evaluated the membrane-affinity column kit performance. A primary evaluation of the exoRNeasy serum/plasma Maxi Kit (QIAGEN) showed its ability to isolate equal or higher amounts of EV and EV-derived RNA in comparison to an optimized DU protocol [35]. In contrast, a study that compared ExoEasy kit (QIAGEN) performance with SEC-qEV, which is considered a low recovery/high specificity method, found that the kit isolated less EV but comparable RNA yield in plasma samples [37]. Therefore, it is not clear if ExoEasy kit results in higher EV and EV-derived RNA yield when compared to traditional isolation methods.

To clarify this, we assessed ExoEasy kit ability to isolate EV in comparison to a DU protocol in culture supernatant and plasma samples. Also, we analysed EV-derived RNA yield after elimination of non-vesicle RNA in DU and ExoEasy kit samples. Here we present evidence about the utility of ExoEasy kit in EV-derived RNA obtainment.

## Materials and methods

### Cell culture

The diffuse large B cell lymphoma cell line SU-DHL-6 (ATCC, CRL-2959) was used for EV isolation. Cells were maintained in RPMI 1640 medium (No. Cat 30–2001) supplemented with penicillin streptomycin solution 10,000 U/ml at 5% (v/v) from SIGMA (P4333-100ML) and non-heat inactivated FBS at 10% (v/v) from ATCC (No. Cat 30–2020). The cells were incubated at 37°C with 5% CO_2_. For EV harvesting, cells from passages 3–10 were employed. For EV isolation, cultures were supplemented with non-heat inactivated exosome depleted FBS at 10% (v/v) from Thermo Fisher (A2720803).

### Plasma samples

Twenty millilitres of peripheral blood were collected from five healthy donors (2 females and 3 males) between 20–40 years old. None of the donors were taking medication at the time of blood collection. All donors had a 10 h fasting. Blood collection was done according to the International Society for Extracellular Vesicles (ISEV) guidelines [1]. The blood was collected in tubes with acid citrate dextrose (ACD) anticoagulant. Briefly, peripheral blood was centrifuged at 2500 g for 15 min at RT (x2). The plasma obtained was divided in 4 ml aliquots and then stored frozen at -20°C. These aliquots were later employed for EVs isolation.

### Ethics statement

This study was approved by the Ethics Committee at Instituto Nacional de Ciencias Medicas y Nutricion Salvador Zubiran in Mexico City. The protocol reference number is HEM-2284-17/22-1. A written informed consent was obtained from all participants. The sample collection and treatment were carried out in accordance with the approved guidelines.

### EV isolation

**Fig 1:**
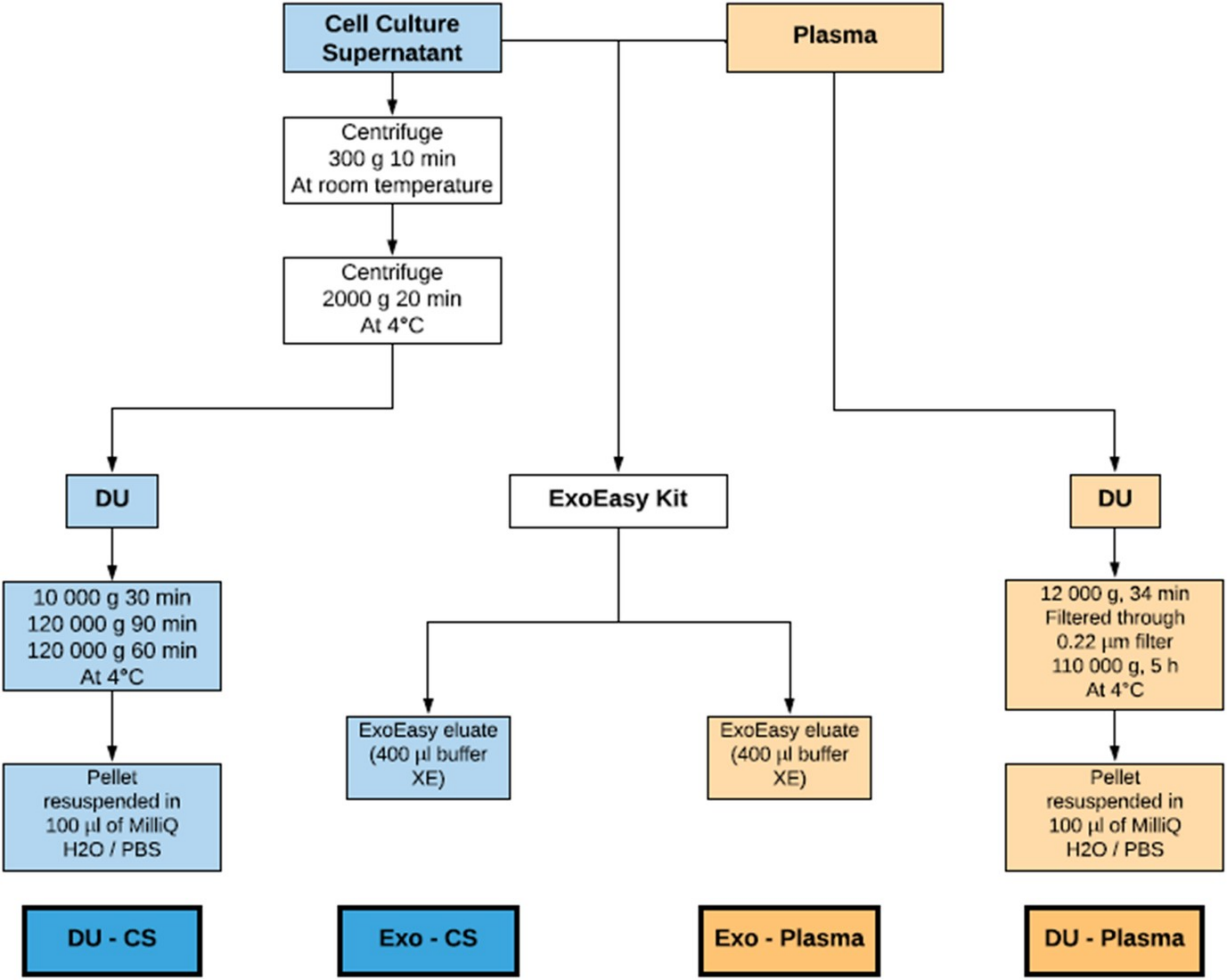
Schematic summary of EV isolation. EV were isolated from culture supernatant and human plasma using a differential ultracentrifugation protocol and the ExoEasy kit according to manufacturer’s instructions.

Culture supernatant was collected from 2x10^7^ cells grown in T75 flasks. Twenty-four hours before EVs harvesting, cell cultures were washed once with PBS followed by a 24 h incubation with 25 ml of fresh culture medium containing 10% (v/v) of exosome depleted FBS. EV isolation from culture supernatants was done according to the protocol of Thery et al. [33]. Briefly, culture supernatant was centrifuged at 300 g for 10 minutes at RT to remove detached cells. Then, supernatant was centrifuged at 2,000 g for 20 min at 4°C. Next, collected supernatant was centrifuged in an Optima XPN Ultracentrifuge (Beckman, Coulter) at 10,000 g with a Type 90 Ti rotor for 30 min at 4°C. After that, supernatant was centrifuged at 120,000 g for 90 min at 4°C to pellet the exosome-enriched fraction. Next, the pellet was resuspended in 1 ml of filtrated phosphate buffered saline (PBS) and then added with 11 ml of filtrated PBS in order to fill up the tube. The samples were centrifuged at 120,000 g for 60 min at 4°C. The supernatant was carefully removed, and the exosome-enriched pellets were resuspended in 100 μl of filtrated PBS or miliQ water depending on downstream experiments (Fig 1).

For plasma, two millilitres were diluted with an equal volume of filtrated PBS. The diluted plasma was centrifuged at 12,000 g for 34 min at 4°C in an Optima XPN ultracentrifuge with a Type 90 Ti rotor (Beckman Coulter, US) to remove dead cells and large vesicles. The supernatant was passed through a 0.22 μm filter. Then, exosome-enriched fraction was pelleted with DU at 110,000 g for 5 hours at 4°C. The supernatant was aspirated leaving the pellet at the bottom of the tube. The final pellet was resuspended in 100 μl of PBS or miliQ water depending on downstream experiments (Fig 1).

### ExoEasy kit

Isolation of EVs with ExoEasy Maxi Kit (Qiagen, USA) was performed according to the manufacturer’s protocol. Briefly, 25 ml of culture supernatant (previously centrifuged at 300 g for 10 min at RT) or 2 ml of plasma, were passed through 0.8 μm filter and mixed with buffer XBP (1:1). Next, the samples were transferred into the ExoEasy membrane affinity column and then centrifuged at 500 x g for 1 min at RT. After discarding the flow through, 10 ml of Buffer XWP was added to the column and centrifuged at 5000 x g for 5 min at RT. After the flow through was discarded, washing buffer was added to the column to remove non-specifically retained material. Then, 400 μl of buffer XE was added directly to the column in order to eluate the EVs. Final eluates were stored at 4°C and used on the same day in downstream experiments (Fig 1).

### Nanoparticle tracking analysis (NTA)

The concentration and size of particles was evaluated with NTA using a NanoSight NS300 (Malvern Instruments, UK). The starting material was diluted with filtrated PBS to obtain 30–60 particles per frame and then loaded manually into the chamber with a syringe. Three 30-second videos were recorded for each sample using a camera level of 13 and detection threshold set at 3. The temperature was monitored throughout the measurements. The videos recorded for each sample were analysed with NTA 3.2 Dev Build 3.2.16 software. The NanoSight system was calibrated with polystyrene latex microbeads of 100 and 200 nm.

### Transmission electron microscopy (TEM)

A sample of EV suspension (10 μl) was placed onto a 200-mesh copper grid with carbon coated formvar film and incubated for 3 min. Excess liquid was removed by blotting. The grid was briefly placed on 10 μl of 2% uranyl acetate (w/v) followed by blotting to remove excess liquid. The grid was observed and photographed under a transmission electron microscope Tecnai Spirit Bio TWIN transmission electron microscope (Hillsboro, OR, USA).

### Western blot analysis

DU and Exo samples were added to 5x RIPA buffer to a final concentration of 1x RIPA buffer in the samples. The lysates were vortexed, incubated on ice for 5 min and subsequently centrifuged at 14,000 rpm for 10 min at 4°C. The soluble fraction was recovered, and protein concentration was measured by the Bradford assay (BioRad). The lysates were added to 5xSB (0.05 mM Tris pH = 6.8, 2% (v/v) SDS, 0.1% Glycerol, bromophenol blue) to a final concentration of 1xSB. The samples were vortexed and subsequently boiled for 5 min at 95°C. Calculations were made in order to load 10 μg and 2 μg of total protein from plasma and culture supernatant lysates, respectively. All samples were loaded into a 7.5% SDS PAGE gel. The following antibodies were used for analysis: CD9 (SC-13118), CD63 (SC-5275), Flotillin-1 (CS-18634), Calnexin (C02-2679S), Albumin (C02-4929S), Apo A-1 (SC-376818). For silver staining, gels were fixed with a trichloroacetic acid solution (20% w/v) and then with a methanol:acetic acid (40:10 v/v) solution. Next, sensitization was done with a glutaraldehyde solution. Then, impregnation was performed with a silver nitrate solution and developed with a formaldehyde and citric acid solution.

### RNA extraction and quantitation

DU and Exo samples were incubated with proteinase K (20 mg/ml) for 10 minutes at 56°C. Next, RNAse A was added at 0.02 mg/ml for 20 min at 37°C. For RNA extraction, Qiazol was added to the sample (10:1) followed by vortex and incubation at RT for 5 min. Then, chloroform was added to the sample (5:1) followed by vortex and room temperature incubation for 2 min. Samples were centrifuged at 12,000 g for 15 min at 4°C. The aqueous phase containing the RNA was transferred to a new tube with ammonium acetate (0.5 M), glycogen (150 μg/ml) and isopropanol. The mixture was incubated for 16 h at -20°C. On the next day, samples were centrifuged at 10,000 rpm for 30 min at 4°C. Next, the supernatant was discarded, and the pellet was rinsed twice with 80% ethanol. Finally, the pellet was left to dry for 5 min and then resuspended in 10–20 μl of MilliQ water. Then samples were incubated at 60°C for 15 min and once they cooled, these were stored at -80°C.

The RNA yield and size were analysed by chip electrophoresis. For Agilent RNA 6000 Nano kit and small RNA kit, 1 μl of RNA was added to the chip and then analysis was performed according to manufacturer’s protocol.

### Statistics

Comparison between any two groups was performed by the student’s t test. P values of <0.05 were considered statistically significant.

## Results

### EV were isolated from culture supernatant by DU and ExoEasy kit

**Fig 2:**
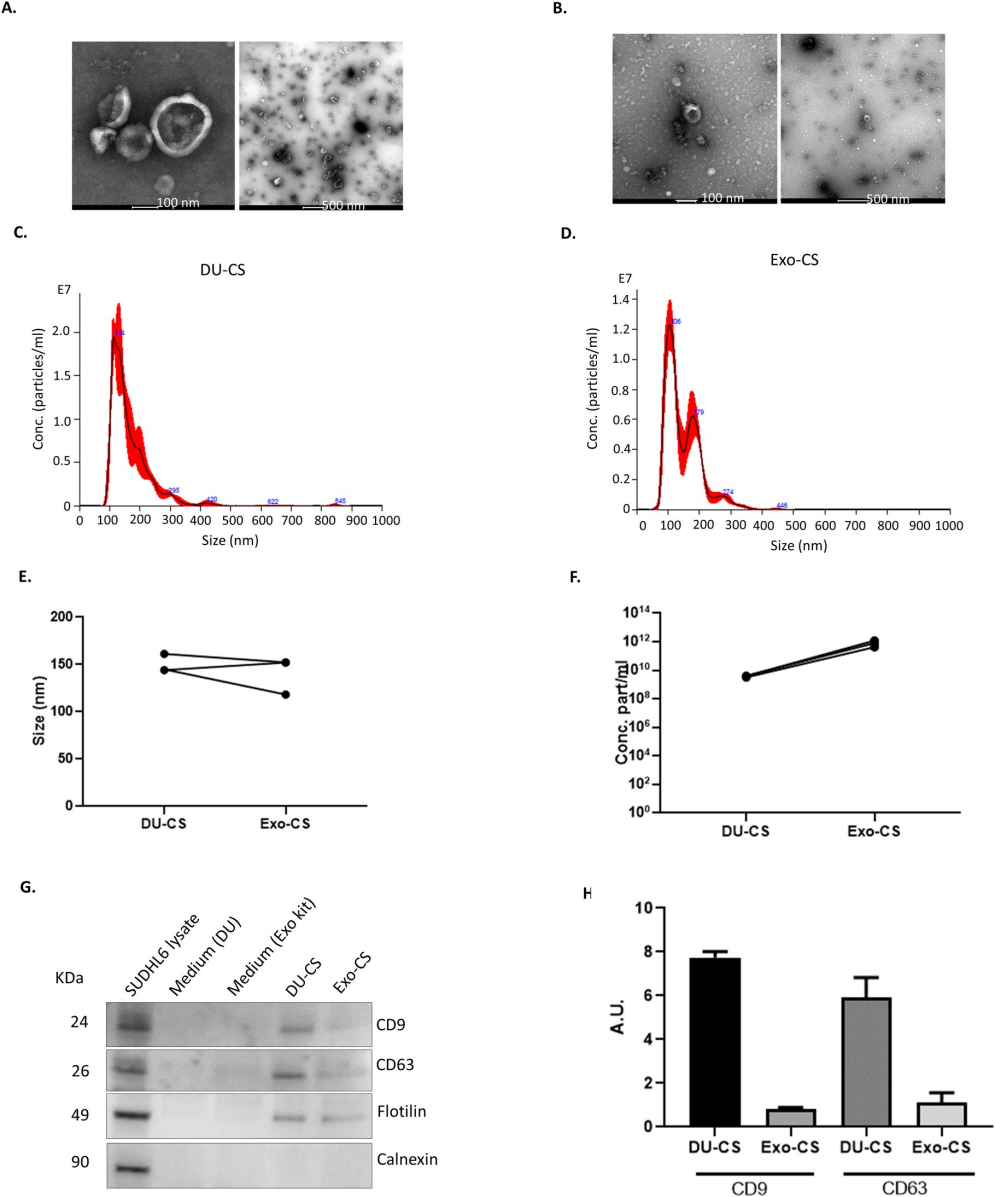
Characterization of EVs isolated from culture supernatant. (A) TEM images corresponding to DU-CS samples. The left panel is a close-up image of 100 nm scale and the right panel is a wide-field image of 500 nm scale. (B) TEM images corresponding to Exo-CS samples. The left panel is a close-up image of 100 nm scale while the right panel is a wide-field image of 500 nm scale. Images are representative of two independent experiments. (C) A representative histogram of DU-CS samples and (D) Exo-CS samples showing the frequency of particle size. (E) The median size value and (F) particle concentration of both DU-CS and Exo-CS samples for three independent experiments is shown. Each sample was analysed in triplicate. (G) Protein lysates of SUDHL6 cells, DU-CS, Exo-CS, Medium DU (negative control) and Medium Exokit (negative control) were analysed by immunoblot for CD9, CD63, Flotilin and Calnexin. Representative immunoblots of two independent experiments are shown. (H) Protein levels were quantitated using Image J analysis of Western Blot signals. Results are expressed in arbitrary units (A.U.).

We investigated the presence of EV in DU-CS and Exo-CS samples by electron microscopy. Some representative close-up and wide-field TEM images of DU-CS (Fig 2A) and Exo-CS (Fig 2B) samples are shown. Both DU-CS and Exo-CS samples revealed the presence of cup-shaped structures, a commonly applied morphological definition for extracellular vesicles [38]. However, a greater number of EV-like structures were observed in DU-CS samples in comparison to Exo-CS samples. This observation can be corroborated in the wide-field images. The EV-like structures found in DU-CS had sizes ranging from 30 nm to 300 nm while those in the Exo-CS samples had a size between 100 and 200 nm.

To directly measure the size and concentration of nanoparticles present in both DU-CS and Exo-CS samples, NTA was performed. A representative histogram of DU-CS and Exo-CS samples displaying the frequency of particle size are shown (Fig 2C and 2D). Although both populations of nanoparticles had a similar size (DU-CS median = 150 ± 5.5 nm and Exo-CS median = 140 ± 11.2 nm); there was a significantly greater concentration of nanoparticles in Exo-CS sample (8x10^11^ ± 2.2x10^11^ particles/ml) in comparison to DU-CS sample (3.7x10^9^ ± 2.8x10^8^ particles/ml) (Fig 2E and 2F).

Then we investigated whether these structures expressed EV markers such as tetraspanins CD9, CD63 and membrane-associated protein Flotillin. To do this, DU-CS and Exo-CS samples were lysed and quantified by immunoblotting. The EV markers CD9, CD63 and Flotillin were expressed in SUDHL6 cells lysate as well as in DU-CS and Exo-CS samples. (Fig 2G). In contrast, these proteins were absent from DU-derived EVs and Exo-derived EVs both isolated from medium (10% commercial exosome depleted FBS), that had not been exposed to cells (negative control). Importantly, the intensity of the bands corresponding to CD9, CD63 and Flotillin was increased in DU-CS in comparison to Exo-CS as shown by the quantitation (Fig 2H). Both DU-CS and Exo-CS showed the absence of calnexin, a chaperone associated to endoplasmic reticulum that is indicative of cellular contaminants [39]. Overall data shows that Exo-CS had a higher concentration of particles in comparison to DU-CS samples; however, the latter had a greater intensity of EV markers signal and an augmented amount of EV-like structures.

### EV were isolated from plasma by DU and ExoEasy kit

**Fig 3:**
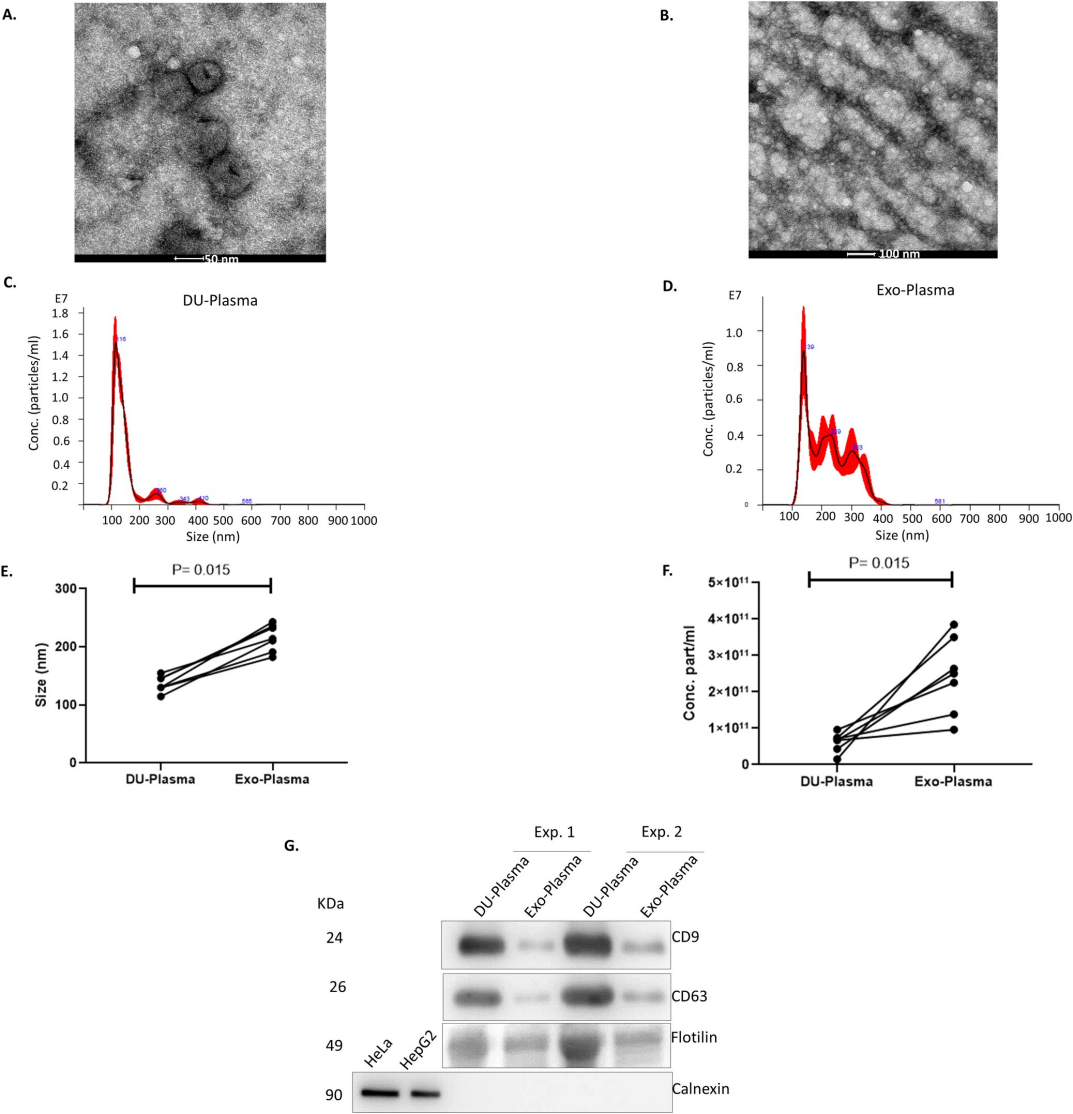
Characterization of EVs isolated from human plasma. (A) TEM image of DU-Plasma sample (50 nm-scale) and (B) TEM image of Exo-Plasma sample (100 nm-scale) are shown. DU-Plasma and Exo-Plasma isolated EVs were analysed by NTA. (C) A representative histogram of DU-Plasma samples and (D) Exo-Plasma samples displaying the frequency of particle size are shown. (E) The median size value and (F) particle concentration of both DU-Plasma and Exo-Plasma samples for seven independent experiments is shown. Each sample was analysed in triplicate. (G) Protein lysates of DU-Plasma and Exo-Plasma were analysed by immunoblot for CD9, CD63, Flotillin and Calnexin. A representative immunoblot of four independent experiments is shown.

As previously done for CS samples, TEM images were taken from both DU-Plasma and Exo-Plasma samples. Although some EV-like structures with a cup-shaped appearance were observed in DU-Plasma samples, an amorphous material, probably derived from the co-isolated proteins and lipids that constitute the plasma as suggested by Webber et al. 2013, was also present (Fig 3A) [40]. In the case of Exo-plasma samples, we could not localize any EV-like structure due to the presence of amorphous material and probably because EV-like structures were less abundant than in DU-Plasma samples (Fig 3B).

Then we studied the size and concentration of nanoparticles in DU-Plasma and Exo-Plasma samples by NTA. To do this, diluted DU-Plasma and Exo-Plasma samples were analysed by the NanoSight instrument. A representative histogram of DU-Plasma (Fig 3C) and Exo-Plasma (Fig 3D) samples displaying the frequency of particle size (nm) is shown. Both samples contained a predominant population of particles measuring about 100 nm in size; however, Exo-Plasma samples had larger populations of approximately 200 and 300 nm in size. Thus, DU-Plasma samples contained on average lower sized-particles (median: 140±5.1 nm) in comparison to Exo-Plasma samples (median: 215±8.7 nm; P = 0.015) (Fig 3E). The number of particles present in Exo-Plasma samples was almost 4-fold greater than that obtained in DU-Plasma samples (P = 0.015) (Fig 3F).

The expression of EV markers was analysed in DU-Plasma and Exo-Plasma samples. Although both samples expressed tetraspanins CD9, CD63 and membrane-associated protein Flotillin, higher intensity bands were shown in DU-Plasma samples (Fig 3G). Cellular contaminants were absent from all samples as shown by calnexin.

**Fig 4:**
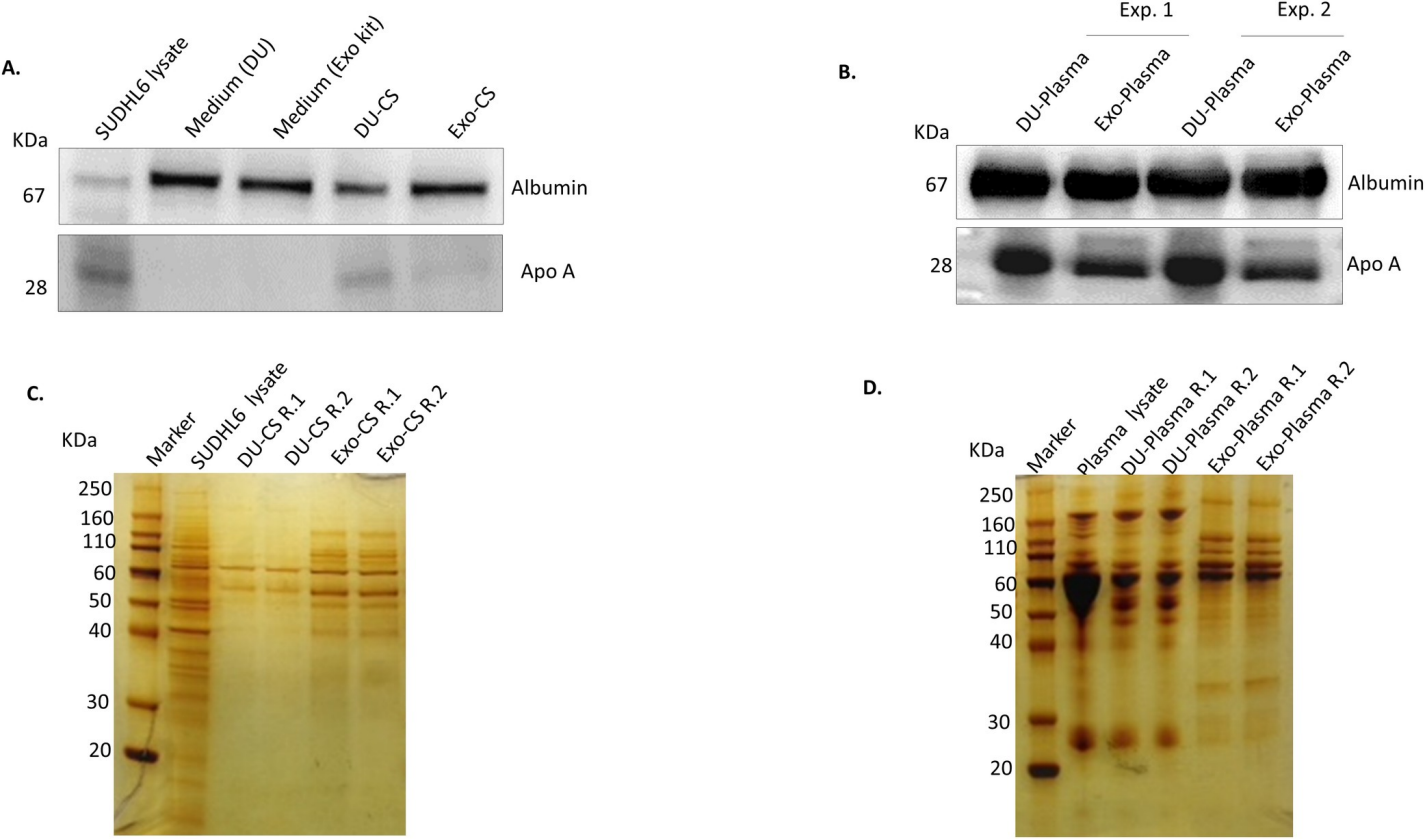
Reduced amount of HDL is co-isolated in ExoEasy kit preparations. (A) Protein lysates of DU-CS; Exo-CS and (B) DU-Plasma; Exo-Plasma were analysed by immunoblot for Albumin and Apolipoprotein A-I. (C) SDS-PAGE gel loaded with SUDHL6 lysate, DU-CS and Exo-CS lysates was stained with silver nitrate. Two replicates of the same CS sample were loaded side by side (R.1 and R.2). (D) SDS-PAGE gel loaded with whole human plasma lysate, DU-Plasma and Exo-Plasma lysates was stained with silver nitrate. Two replicates of the same samples were loaded side by side (R.1 and R.2).

Therefore, Exo-Plasma samples had a larger number of particles but a lower amount of EV-like structures and EV markers when compared to DU-Plasma samples. Our data shows that the extra particles detected by NTA in Exo-Plasma samples do not come from EV resuspension buffer (S1 Fig) nor from co-isolated lipids (Fig 4A and 4B).

### Non-vesicular elements co-isolated with EV by DU and ExoEasy kit

EV have been reported to co-isolate with albumin, protein complexes and lipoproteins [41]. Therefore, we detected by immunoblotting the presence of albumin and Apolipoprotein A-I. The latter accounts for almost 70% of the protein content in HDL (Simonser Circulation Research 2017). Albumin was present in both CS and plasma samples independently of the EV isolation method that was applied: DU or ExoEasy kit. In contrast, Apo A-I had a lower expression in Exo samples in comparison to DU samples (Fig 4A and 4B).

In order to have an overview of the protein repertoire in samples and compare it with that of secreting cells, SDS-PAGE gels were stained with silver nitrate that reacts with NH3 groups from proteins resulting in silver precipitation and thus visualization of proteins. In the first gel, a greater repertoire of proteins from various sizes was observed in SUDHL6 lysates in comparison to DU-CS and Exo-CS samples (Fig 4C). Interestingly, although DU-CS and Exo-CS samples had a smaller protein repertoire, some bands were enriched in these samples when compared to SUDHL6 lysate, thus showing the specificity of the isolated product by DU and ExoMaxi kit. The protein repertoire from CS samples was mainly localized in 40–110 KDa region. In the second gel, human plasma lysate was loaded beside DU-Plasma and Exo-Plasma samples (Fig 4D). The protein repertoire of the plasma sample was similar to that of DU-Plasma samples and in a lesser degree with Exo-Plasma samples. Both DU-Plasma and Exo-Plasma samples showed an enrichment of some protein bands in comparison to the plasma sample, thus suggesting the isolation of a specific content. Due to the similarity of DU-Plasma sample and plasma lysate, it is possible that DU-Plasma samples incorporated more contaminants shared with plasma to the isolated fraction than Exo-Plasma samples.

### Existence of a protected RNA population in EV isolated from culture supernatant

**Fig 5:**
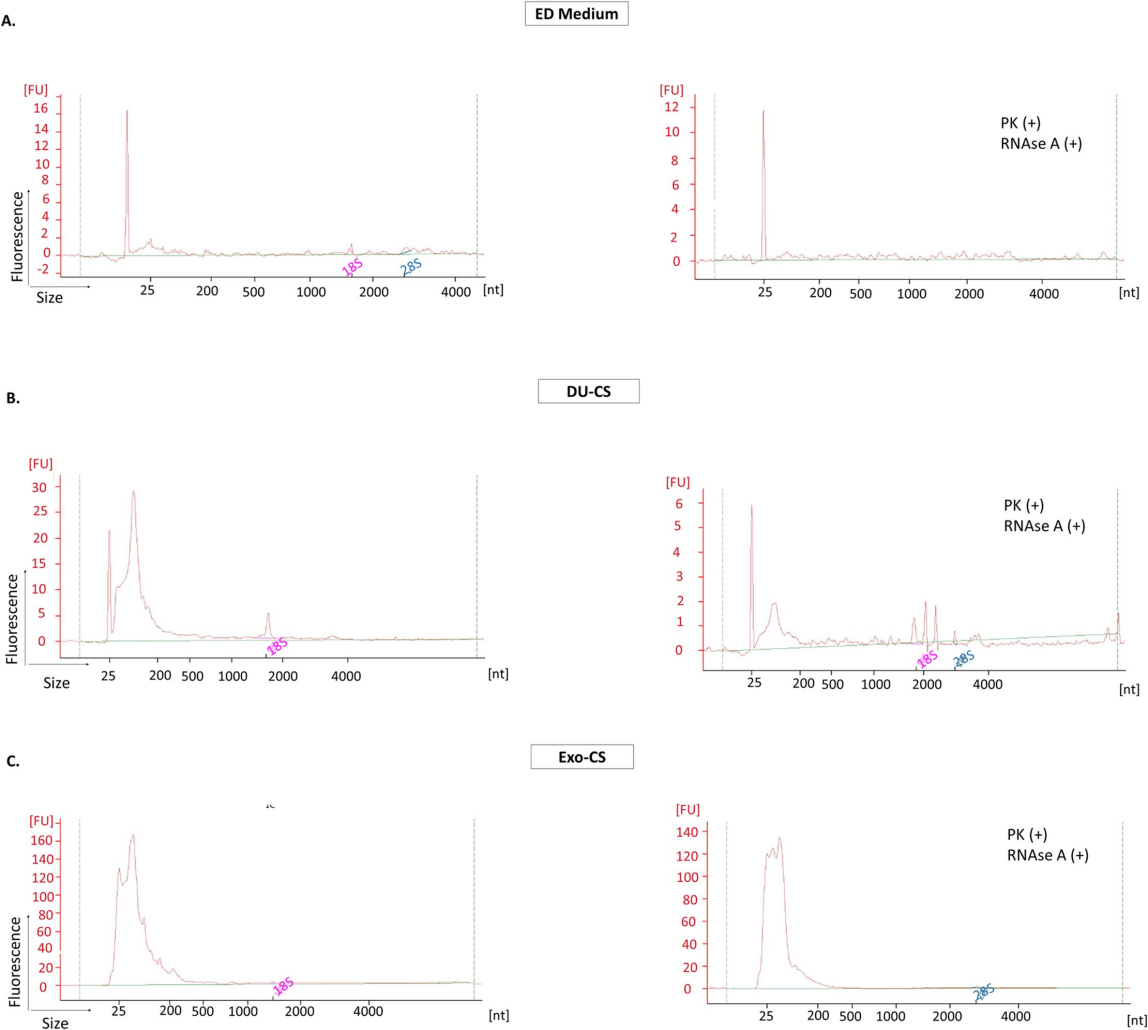
Decrease in total RNA after enzymes treatment in culture supernatant samples. Electropherograms corresponding to **A)** negative control; **B)** DU-CS and **C)** Exo-CS samples before and after proteinase K and RNAse A treatment. All samples were analysed by Agilent RNA 6000 Pico Kit in an Agilent 2100 Bioanalyzer. The electropherograms show the size distribution in nucleotides (nt) and fluorescence intensity (FU) of total RNA. The peak at 25 nt is an internal standard. A representative electropherogram of three and two independent experiments is shown for DU-CS and Exo-CS samples, respectively.

In order to eliminate non-EV RNA from DU-CS and Exo-CS samples, these were treated with proteinase K and subsequently with RNAse A. Then, RNA was extracted and examined by Bioanalyzer equipment using the RNA Pico kit. As negative control, RNA was extracted from DU-derived EVs isolated from medium (10% of commercial exosome depleted FBS), that had not been exposed to cells. As expected, no bovine EV-derived RNA (~200 nt) was present in the samples when measured by bioanalyzer; however, it might be present when analysed by more sensitive techniques (Fig 5A). A representative sample of the electrophoresis histogram from both DU-CS and Exo-CS is shown in Fig 5B and 5C. An enrichment in short RNA (< 200 nt) was observed in both DU-CS and Exo-CS samples as well as the presence of minimum amount of ribosomal 18S (~1900 nt) and 28S (~4200 nt) as previously reported for small vesicles. After enzyme incubation, there was a partial loss of RNA in both DU-CS and Exo-CS; however, an RNA population, potentially enclosed in vesicles, remained in all samples. The protected RNA population was also visualized in the small RNA chip electropherogram (S2 Fig). Overall, these data indicated the presence of non-exosomal RNA in both samples.

### Existence of a protected RNA population in EV isolated from plasma

**Fig 6:**
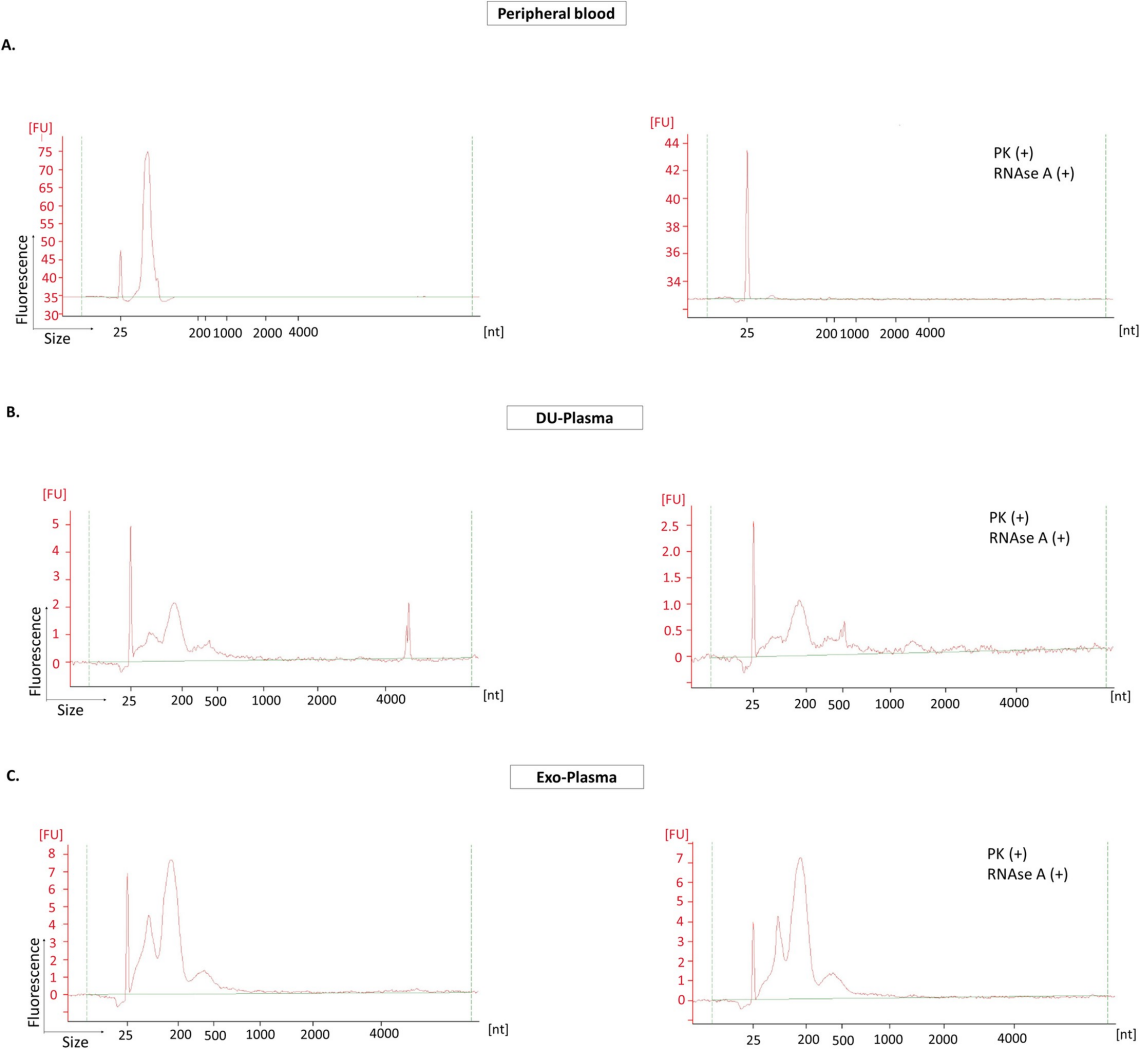
Decrease in total RNA after enzymes treatment in plasma samples. Electropherograms corresponding to **A)** short RNA extracted from peripheral blood; **B)** DU-Plasma and **C)** Exo-Plasma samples before and after proteinase K and RNAse A treatment. All samples were analysed by Agilent RNA 6000 Pico Kit in an Agilent 2100 Bioanalyzer. The electropherograms show the size distribution in nucleotides (nt) and fluorescence intensity (FU) of total RNA. The peak at 25 nt is an internal standard. A representative electropherogram of two independent experiments is shown for DU-Plasma samples. A representative electropherogram of two and one independent experiments for DU-Plasma and Exo-Plasma samples, respectively.

Both DU-Plasma and Exo-Plasma samples were also treated with proteinase K and RNase A before RNA extraction. As a positive control for enzyme conditions, small RNA was isolated from peripheral blood by a combined phenol/column-based isolation method (miRVana, small RNA protocol) and then, treated with proteinase K and RNAseA. As expected, both enzymes resulted in the degradation of all the small RNA present in the sample (Fig 6A). The histograms of both DU-Plasma and Exo-Plasma samples showed an enrichment of small RNA (<200 nt); however, other RNA fragments of longer size (~400 nt) were also present in both samples, probably due to the presence of messenger RNA previously reported in small vesicles (Fig 6A–6C) [42,43]. The enzymes treatment resulted in the degradation of a portion of RNA in both DU-Plasma and Exo-Plasma samples; however, a protected RNA population remained in all samples, suggesting the presence of non-exosomal RNA in DU-Plasma and Exo-Plasma samples.

### Increased yield of protected RNA in ExoMaxi Kit products

**Fig 7:**
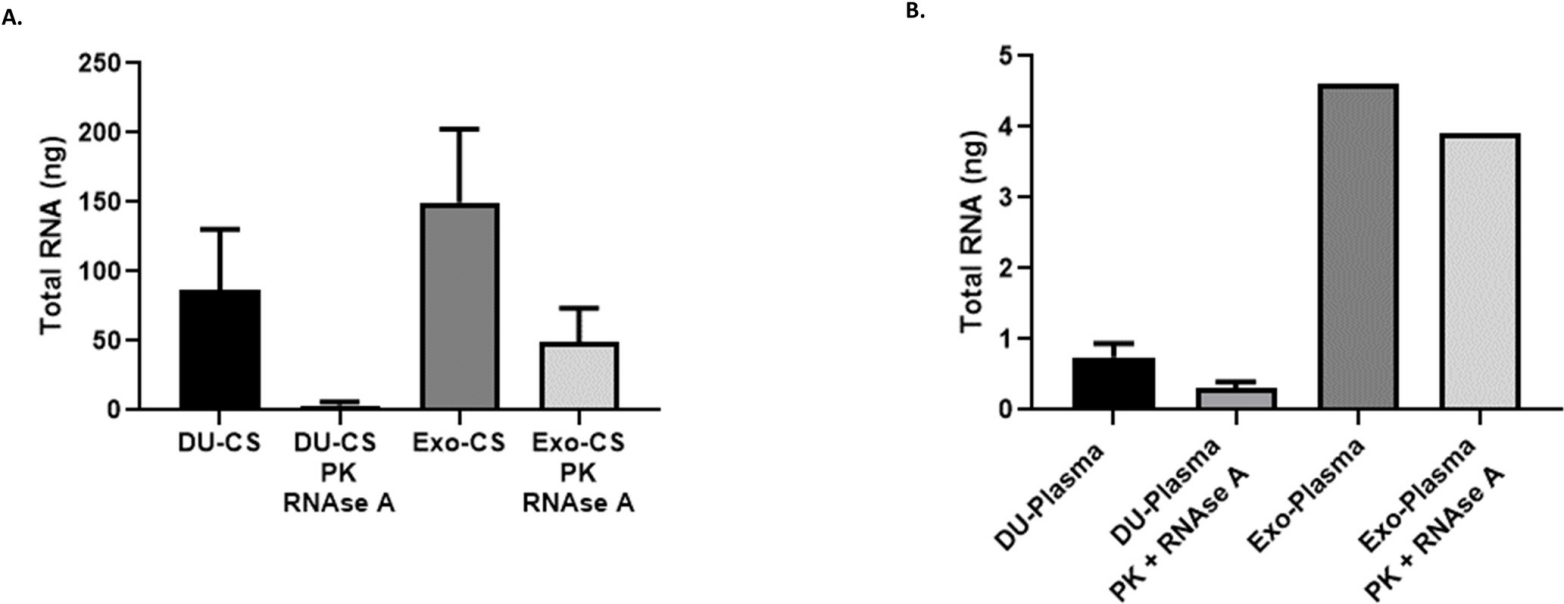
Exo samples have a greater RNA yield even after enzymatic treatment. RNA quantitation in (A) DU-CS; Exo-CS samples and (B) DU-Plasma; Exo-Plasma samples, before and after proteinase K and RNAse A treatment. Each bar represents mean values ± SD of total RNA (ng) obtained in independent experiments.

Total RNA extracted from DU-CS samples (mean: 86.8 ng) was lower than Exo-CS samples (mean: 149.8 ng). After enzymatic treatment, a similar tendency was observed. Exo-CS samples had a greater RNA recovery (mean: 49 ng) in comparison to DU-CS samples (mean: 3.3 ng) (Fig 7A).

When comparing RNA yield in DU-Plasma and Exo-Plasma, there was a higher yield of total RNA in the latter case. After enzymatic treatment, Exo-Plasma still had the highest RNA yield (mean: 3.9 ng) in comparison to DU-Plasma (0.2 ng) (Fig 7B). This was also corroborated by MicroQubit assay where DU-Plasma samples showed a lower RNA yield (1.4 ng) in comparison to Exo-Plasma samples without enzymatic treatment (49.2 ± 2.3 ng) or after enzymes addition (31.7 ± 5.2ng) (S3 Fig). Therefore, the highest protected RNA yield in both culture supernatant and plasma samples was obtained by ExoEasy kit.

## Discussion

It is not clear how efficient is the performance of membrane affinity column kits in terms of EV recovery and EV-derived RNA yield. Thus, we compared the characteristics of ExoEasy kit derived EV as well as their RNA content to those obtained by DU when processing culture supernatant and plasma samples.

All samples used the same starting CS or plasma sample as well as the same RNA extraction protocol in order to make possible the comparison between ExoEasy Maxi kit and DU. Our data showed that Exo-CS samples exhibited a scarce number of EV-like structures by TEM in comparison to DU-CS samples. In addition, a lower expression of EV markers CD63, CD9 and Flotillin was detected by immunoblotting in Exo samples (Exo-CS and Exo-Plasma) when compared to DU samples (DU-CS and DU-Plasma). These data suggested a higher EV recovery in DU samples when compared to Exo samples. However, a greater particle concentration detected by NTA in Exo samples as well as a higher RNA yield (2.3 ng/ml plasma; 149 ng/2x10^7^ cells), did not reflect the apparently greater EV recovery by DU protocols.

In order to understand this contradiction, we decided to investigate if the greater RNA yield in Exo samples was related to the presence of non-vesicular RNA. Previous studies have shown that RNA from the EV fraction can be completely degraded by treating with a mild detergent, proteinase K and RNAse A [44]. Because we were interested in degrading the RNA bound to Ago 2 and other proteins but not the vesicular RNA, we only treated with proteinase K and RNAse A before RNA extraction. Previous reports examining ExoEasy kit performance did not use pre-treatment with proteinase K and RNAse A [35,37]. Our data showed that treatment with the enzyme combination resulted in a reduction of RNA in both Exo and DU samples in comparison to untreated samples. This suggested that both methods had non-exosomal RNA contamination. However, a protected RNA population remained in every sample after enzymatic treatment, suggesting that this might be contained within EV. Importantly, after enzymatic treatment, the protected RNA population was still higher in Exo samples.

Therefore, a plausible explanation for the fact that a lower protected RNA yield was found in DU samples while having an enrichment of EV-like structures and EV markers, might be related to the technique itself. Some authors have proposed that, given the great centrifugal force (100 000 g) required in order to obtain EV, DU is associated with increased damage to the integrity of EV structure when compared with other isolation methods [45,46]. This has been linked to a lower EV recovery [34,47].

It is possible that DU is a more aggressive procedure than affinity column thus resulting in the breaking of some EV. This might lead to the leakage of EV-derived RNA which will be susceptible to the action of proteinase K and RNAse A, thus resulting in a lower RNA yield. In this same line of reasoning, the fragments of damaged EV still retained CD63, CD9 and Flotillin expression that could be detected by immunoblotting, thus giving a higher signal in DU samples. However, the fragments of broken EV, which will probably have a size shorter than 60 nm (detection limit of NS300) [48–50], will become undetectable by the Nanosight thus resulting in a higher particle number for Exo samples. Therefore, it may be that the great majority of the particles detected by NTA in Exo-Plasma are EV; however, because most of them are intact, the particle count is higher than in DU samples.

In terms of co-isolated contaminants, we showed by immunoblotting and fluorescent gel staining that albumin is present in both Exo samples and DU samples. Apo A-I which is a major component of high-density lipoprotein (HDL), was found more concentrated in DU samples than Exo samples, suggesting a higher amount of HDL in DU preparations. This is important as HDL has been found to transport microRNAs [51]. ApoB 48 which is a major component of chylomicrons was present in DU samples only.

Based on the results reported in this study, ExoEasy kit might be a more delicate technique which leads to a higher RNA yield in comparison to DU. We proposed the incubation of the ExoEasy kit eluate with proteinase K and RNAse A before RNA extraction as we showed the presence of non-EV RNA in the ExoEasy kit product. The yield of protected RNA obtained by ExoEasy kit in both CS and plasma samples has an acceptable concentration for library preparation as well as having low contamination with lipoproteins. Therefore, ExoEasy kit is a good option for EV-derived RNA workflows.

## Supporting information

S1 Fig Blank samples have a lower particle size and concentration in comparison to plasma samples. (A) Particle concentration measured by NTA in filtered PBS, diluted DU-Plasma sample (1:150) and undiluted DU-Plasma sample. (B) Particle concentration measured by NTA in buffer XE, diluted Exo-Plasma sample (1:600) and undiluted Exo-CS. (C) Median size value and concentration of particles found in PBS and buffer XE.
(TIF)

S2 Fig Protected RNA population includes sequences with a similar size to microRNAs. Electropherograms corresponding to DU-CS (A-C) and Exo-CS samples (B-D) before and after proteinase K and RNAse A treatment. All samples were analysed by Agilent small RNA Kit in an Agilent 2100 Bioanalyzer (4–150 nt). The electropherograms show the size distribution in nucleotides (nt) and fluorescence intensity (FU) of small RNA. The peak at 4 nt is an internal standard. The region delimited from 10–40 nt correspond to miRNA-like species. A representative electropherogram of two independent experiments is shown.
(TIF)

S3 Fig Higher concentration of protected RNA in Exo-Plasma samples. Quantitation of small RNA by MicroQubit assay in Exo-Plasma samples before and after Proteinase K and RNAse A treatment. Each bar of Exo-Plasma and Exo-Plasma + RA + PK represent the mean values ± SD of total RNA (ng) obtained in three independent experiments.
(TIF)

S1 File (DOCX)

S2 File (XLSX)

S1 Raw images (TIF)
