# Supplementary figures and images for: Analysis of RNA yield in extracellular vesicles isolated by membrane affinity column and differential ultracentrifugation
Source: PLoS One. 2020 Nov 6;15(11):e0238545. doi: 10.1371/journal.pone.0238545 (PMC7647092; doi:10.1371/journal.pone.0238545)

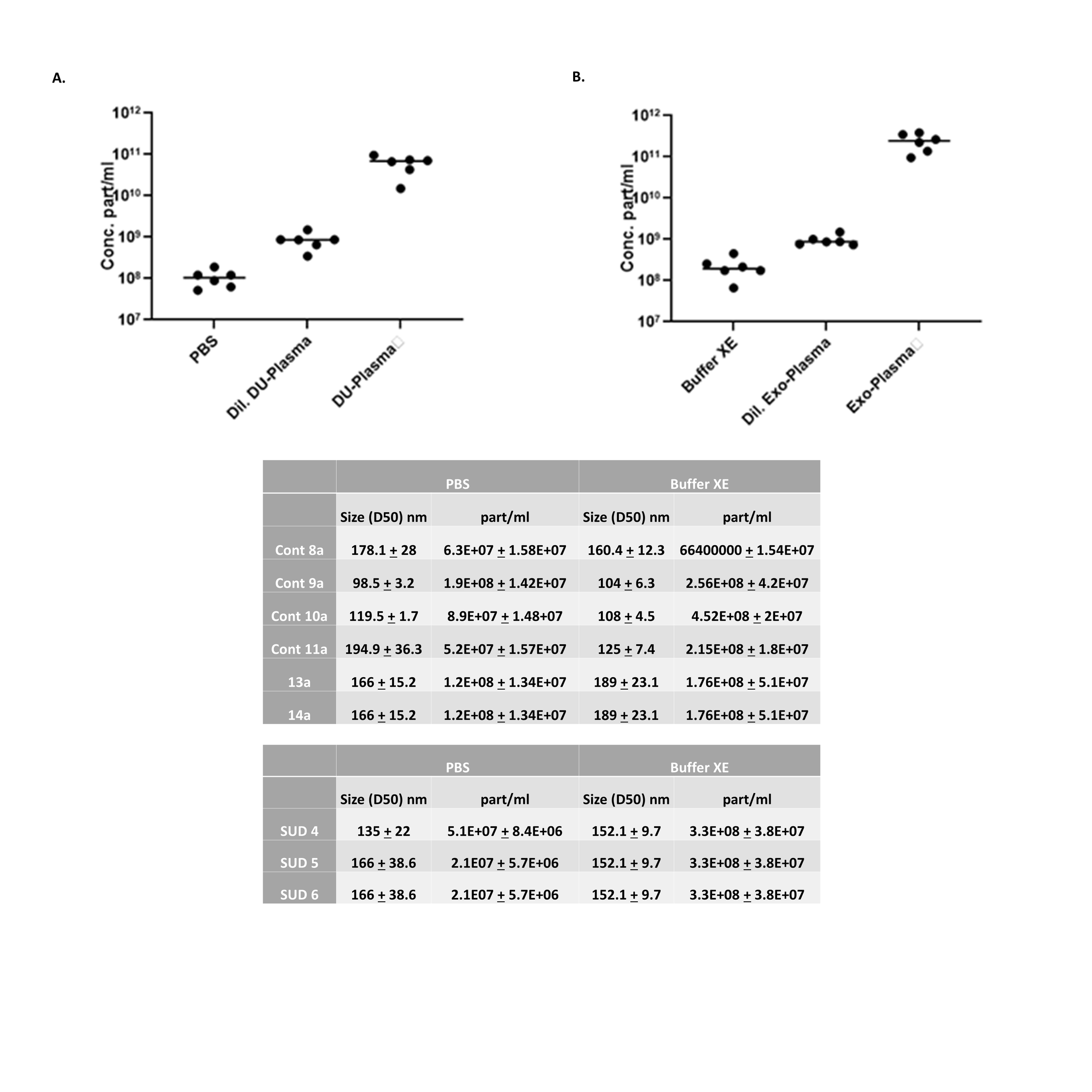

Supplement: S1 Fig — (A) Particle concentration measured by NTA in filtered PBS, diluted DU-Plasma sample (1:150) and undiluted DU-Plasma sample. (B) Particle concentration measured by NTA in buffer XE, diluted Exo-Plasma sample (1:600) and undiluted Exo-CS. (C) Median size value and concentration of particles found in PBS and buffer XE. (TIF) [file pone.0238545.s001.tif]

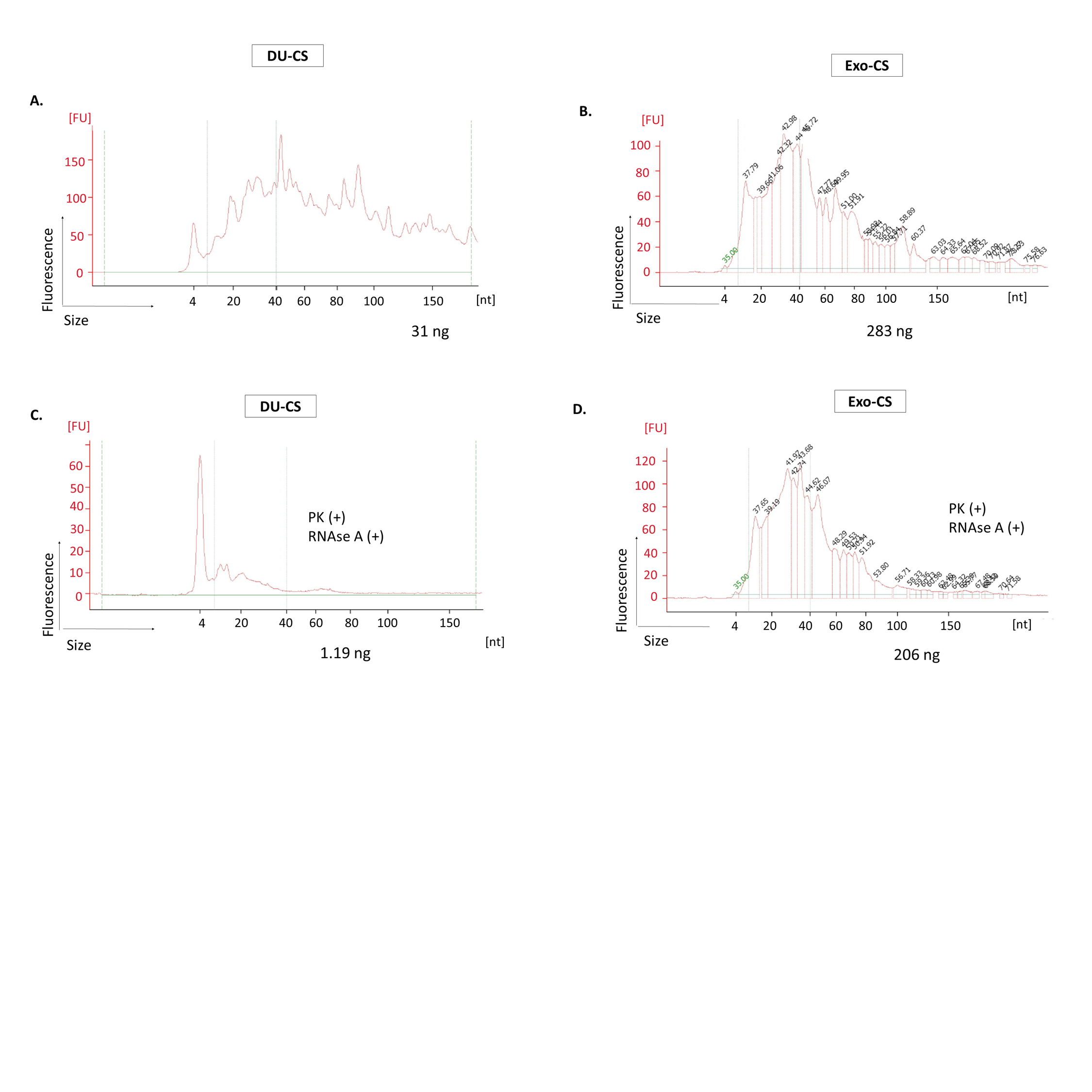

Supplement: S2 Fig — Electropherograms corresponding to DU-CS (A-C) and Exo-CS samples (B-D) before and after proteinase K and RNAse A treatment. All samples were analysed by Agilent small RNA Kit in an Agilent 2100 Bioanalyzer (4–150 nt). The electropherograms show the size distribution in nucleotides (nt) and fluorescence intensity (FU) of small RNA. The peak at 4 nt is an internal standard. The region delimited from 10–40 nt correspond to miRNA-like species. A representative electropherogram of two independent experiments is shown. (TIF) [file pone.0238545.s002.tif]

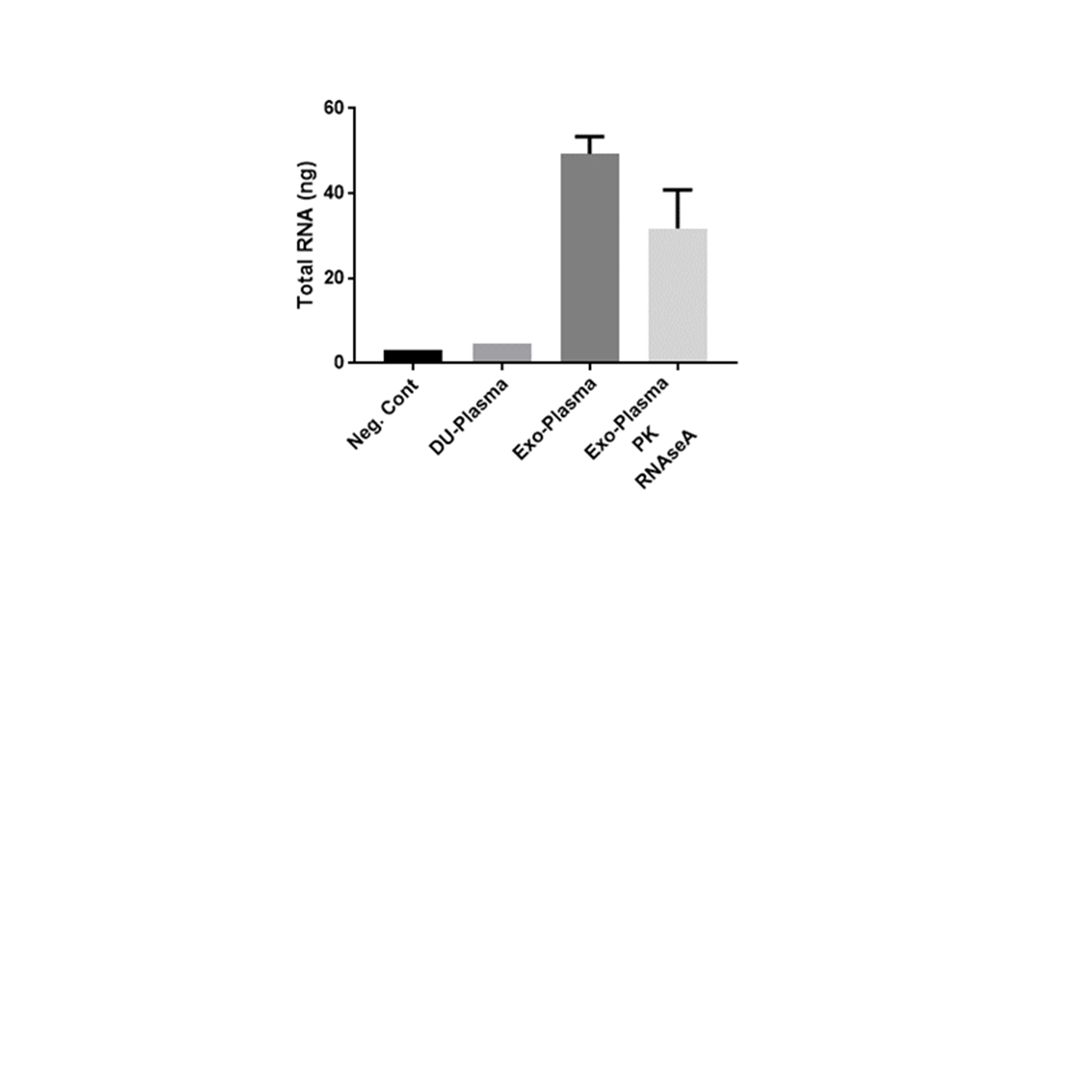

Supplement: S3 Fig — Quantitation of small RNA by MicroQubit assay in Exo-Plasma samples before and after Proteinase K and RNAse A treatment. Each bar of Exo-Plasma and Exo-Plasma + RA + PK represent the mean values ± SD of total RNA (ng) obtained in three independent experiments. (TIF) [file pone.0238545.s003.tif]

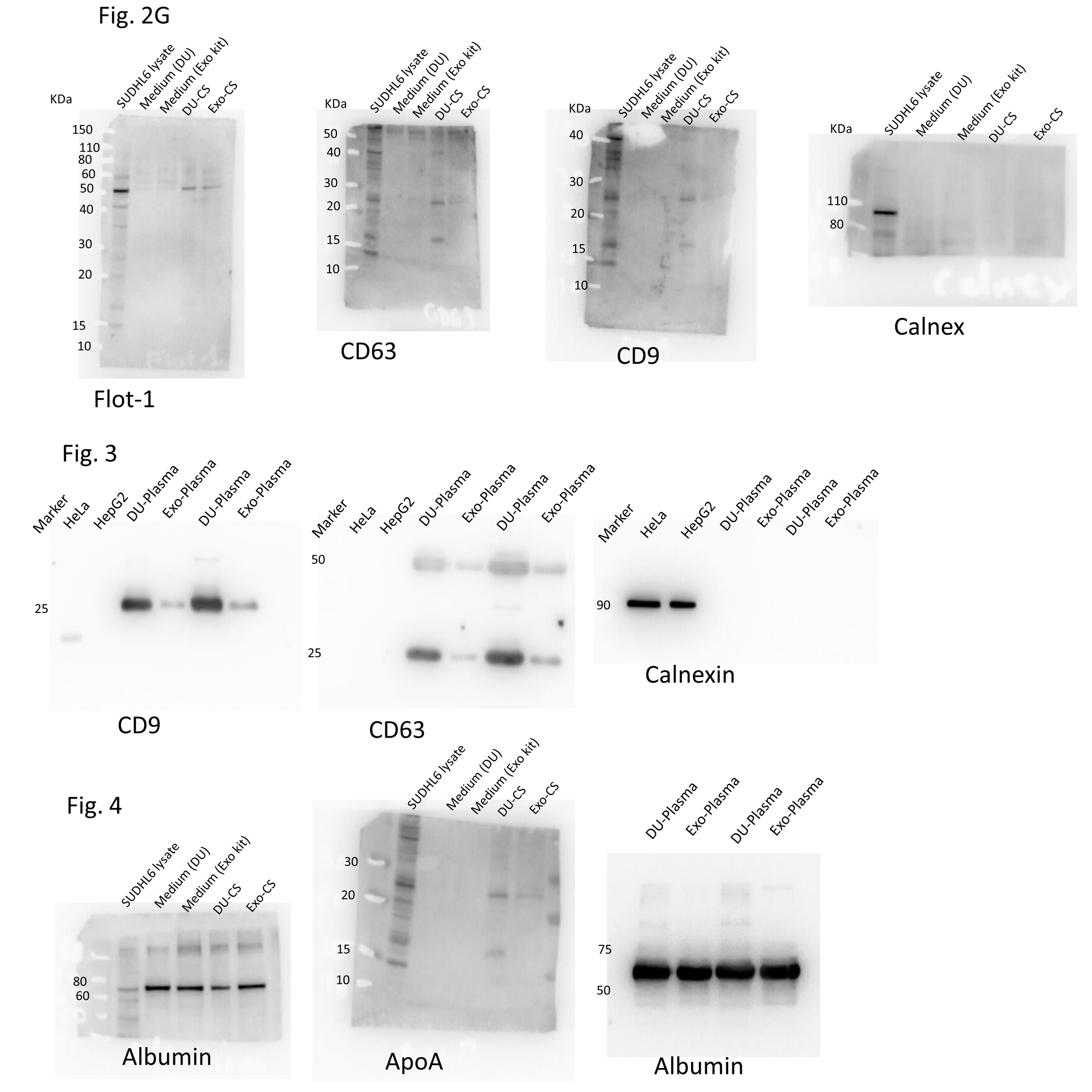

Supplement: S1 Raw images — (TIF) [file pone.0238545.s006.tif]
