# Supplementary material for: Analysis of RNA yield in extracellular vesicles isolated by membrane affinity column and differential ultracentrifugation
Source: PLoS One. 2020 Nov 6;15(11):e0238545. doi: 10.1371/journal.pone.0238545 (PMC7647092; doi:10.1371/journal.pone.0238545)
Supplement: S1 File — (DOCX) [file pone.0238545.s004.docx]

EV isolation from culture supernatant by DU

Culture supernatant was collected from 2x10^7^ cells grown in T75 flasks. Twenty-four hours before EVs harvesting, cell cultures were washed once with PBS followed by a 24 h incubation with 25 ml of fresh culture medium containing 10% (v/v) of exosome depleted FBS. EV isolation from culture supernatants was done according to the protocol of Thery et al. [28]. Briefly, 25 ml of culture supernatant was divided into two volumes of 12.5 ml in order to be placed into propylene tubes with a 13.5 ml volume capacity. Each tube was centrifuged at 300 g for 10 minutes at RT to remove detached cells. Then, each supernatant was centrifuged at 2,000 g for 20 min at 4°C. Next, each collected supernatant was centrifuged in an Optima XPN Ultracentrifuge (Beckman, Coulter) at 10,000 g with a Type 90 Ti rotor for 30 min at 4°C. After that, each supernatant was centrifuged at 120,000 g for 90 min at 4°C to pellet the exosome-enriched fraction. Next, each pellet was resuspended in 1 ml of filtrated phosphate buffered saline (PBS) and then added with 11 ml of filtrated PBS in order to fill up the tube. Each sample was centrifuged at 120,000 g for 60 min at 4°C. The supernatant was carefully removed, and each EV-enriched pellet was resuspended in 100 µl of filtrated PBS or miliQ water depending on downstream experiments (Fig 1)

EV isolation from plasma by DU

For plasma, four millilitres were processed. The volume was divided into two equal parts and then diluted with an equal volume of filtrated PBS. Thus, it resulted in two volumes of four millilitres of diluted plasma. Each volume was placed in a propylene tubes with a 13.5 ml volume capacity. In this first centrifugation round, there was no need to fill up the tube to the top. Each diluted plasma was centrifuged at 12,000 g for 34 min at 4°C in an Optima XPN ultracentrifuge with a Type 90 Ti rotor (Beckman Coulter, US) to remove dead cells and large vesicles. Each supernatant was passed through a 0.22 µm filter. Then, each EV-enriched fraction was pelleted with DU at 110,000 g for 5 hours at 4°C. Each supernatant was aspirated leaving the pellet at the bottom of the tube. Each final pellet was resuspended in 100 µl of PBS or miliQ water depending on downstream experiments (Fig 1).

EV isolation from culture supernatant

Isolation of EVs with ExoEasy Maxi Kit (Qiagen, USA) was performed according to the manufacturer’s protocol. Briefly, 25 ml of culture supernatant (previously centrifuged at 300 g for 10 min 130 at RT) were passed through 0.8 µm filter and mixed with buffer XBP (1:1). The total volume (50 ml) was divided into two equal parts that were loaded into two different membrane affinity columns. This in order not to exceed the binding capacity of the column. Next, the samples were centrifuged at 500 x g for 1 min at RT. After discarding the flow through, 10 ml of Buffer XWP was added to each column and centrifuged at 5000 x g for 5 min at RT. After the flow through was discarded, washing buffer was added to each column to remove non-specifically retained material. Then, 400 µl of buffer XE was added directly to each column in order to eluate the EVs. Final eluates were stored at 4°C and used on the same day in downstream experiments (Fig 1)

EV isolation from plasma

Isolation of EVs with ExoEasy Maxi Kit (Qiagen, USA) was performed according to the manufacturer’s protocol. Four mililiters of plasma were processed in each experiment. The volume was divided into two equal parts so that each 2 ml of plasma were passed through 0.8 µm filter and mixed with buffer XBP (1:1). Next, each 4 ml of diluted plasma were transferred into two different ExoEasy membrane affinity column. Both columns were then centrifuged at 500 x g for 1 min at RT. After discarding the flow through, 10 ml of Buffer XWP was added to each column and centrifuged at 5000 x g for 5 min at RT. After the flow through was discarded, washing buffer was added to each column to remove non-specifically retained material. Then, 400 µl of buffer XE were added directly to each column in order to eluate the EVs. Final eluates were stored at 4°C and used on the same day in downstream experiments (Fig 1).

RNA extraction

Both resuspended pellets from DU (100 µl) were added together in order to eliminate variances from individual processing and then the total volume was again divided into two equal parts. This was the same with Exo samples (400 µl). One of the parts for DU and Exo samples was added with enzymes PK (+) RNAseA (+) while the other was added with PBS and labelled PK (-) RNAseA (-). In the case of PK (+) RNAse A (+) samples, they were incubated with proteinase K (20 mg/ml) for 10 minutes at 56°C. Next, RNAse A was added at 0.02 mg/ml for 20 min at 37°C. For RNA extraction, Qiazol was added to the sample (10:1) followed by vortex and incubation at RT for 5 min. Then, chloroform was added to the sample (5:1) followed by vortex and room temperature incubation for 2 min. Samples were centrifuged at 12,000 g for 15 min at 4°C. The aqueous phase containing the RNA was transferred to a new tube with ammonium acetate (0.5 M), glycogen (150 µg/ml) and isopropanol (1.2 fold of the final volume). The mixture was incubated for 16 h at -20°C. On the next day, samples were centrifuged at 10,000 rpm for 30 min at 4°C. Next, the supernatant was discarded, and the pellet was rinsed twice with 80% ethanol. Finally, the pellet was left to dry for 5 min and then resuspended in 10 µl of MilliQ water. Then samples were incubated at 60°C for 15 min and once they cooled, these were stored at -80°C.

RNA quantitation by bioanalyzer

One microliter was taken from the RNA sample in order to measure yield and size by chip electrophoresis. For Agilent RNA 6000 Nano kit and small RNA kit, 1 µl of RNA was added to the chip and then analysis was performed according to manufacturer’s protocol.

RNA quantitation by MicroQubit

Quantitation of RNA by MicroQubit was performed according to the manufacturer’s protocol. Briefly, the mixture of Quant-it reagents and Quant-it buffer was prepared according to instructions. Five microliters of the RNA samples were added to 195 µl of the Quant-it mixture. Then samples were incubated and then analysed in the Qubit 3.0 equipment.
